# Comparison of Three Feline Crossmatch Methods—Tube, Gel Column, and Immunochromatographic Strip

**DOI:** 10.1111/vcp.70039

**Published:** 2025-08-22

**Authors:** Victoria K. DiCiccio, Rebecka S. Hess, Nicole M. Weinstein, Samantha Fromm, Ruth Gonzalez, Kimberly Marryott, Mary Beth Callan

**Affiliations:** ^1^ Department of Clinical Sciences and Advanced Medicine University of Pennsylvania Philadelphia Pennsylvania USA

**Keywords:** alloantibody, antiglobulin, RBC incompatibility, transfusion

## Abstract

**Background:**

A pre‐transfusion blood crossmatch is recommended to ensure RBC compatibility for previously transfused and transfusion‐naïve cats.

**Objectives:**

To compare 3 crossmatch methods and anti‐feline globulin (AFG) enhancement for determining RBC compatibility.

**Methods:**

Prospective study. Major crossmatches were performed using serum from 68 “recipient” cats and RBCs from 44 “donor” cats. Crossmatch methods evaluated include laboratory tube and gel column without and with AFG enhancement and an in‐clinic AFG‐enhanced immunochromatographic strip.

**Results:**

Tube and gel crossmatches were performed for 211 and strip crossmatches for 133 recipient‐donor pairs. RBC incompatibilities were noted in at least 1 crossmatch method for 123 recipient‐donor pairs. For determination of the degree of incompatibility, there was a correlation between standard and AFG‐enhanced crossmatches for both tube and gel (*p* < 0.001), standard tube and gel (*p* = 0.002), and AFG‐enhanced tube and gel (*p* < 0.001). Twelve of 46 and 22 of 113 recipient‐donor pairs deemed incompatible with tube and gel, respectively, had RBC agglutination noted only with the addition of AFG. RBC incompatibilities were noted on 15 strips, 4 of which were type A‐B mismatched. Odds of detecting RBC incompatibility using strip were 2.9 (*p* = 0.001) and 6.1 (*p* < 0.001) times greater with each unit increase in the degree of incompatibility detected by standard and AFG‐enhanced tube, respectively.

**Conclusions:**

The clinical relevance of any observed RBC incompatibilities, other than known A‐B mismatches, is unknown. While the strip crossmatch is simple to perform and interpret, it might not be sufficiently sensitive to detect weaker RBC incompatibilities.

## 1 Introduction

Blood compatibility testing is essential to provide safe and effective blood products to patients. For transfusion‐naïve cats, minimum testing is the determination of AB blood type, as naturally occurring RBC alloantibodies, particularly strong anti‐A alloantibodies present in most type B cats, create a risk for an acute hemolytic transfusion reaction in A‐B‐mismatched transfusions [1, 2]. While it is widely accepted that a pre‐transfusion blood crossmatch is indicated to determine serological compatibility for all cats that received a RBC transfusion ≥ 4 days earlier, there is controversy regarding the need for a blood crossmatch for transfusion‐naïve cats [3, 4, 5, 6, 7, 8, 9, 10, 11, 12].

A novel blood type, *Mik*, was identified during extensive RBC compatibility testing within a feline blood colony, in which naturally occurring alloantibodies against *Mik* were present in transfusion‐naïve donor cats [3]. The clinical relevance of *Mik* alloantibodies was demonstrated when a *Mik*‐negative cat inadvertently received both blood and a kidney from *Mik*‐positive donors, and naturally occurring anti‐*Mik* alloantibodies were identified in the pre‐transfusion recipient plasma during investigation of the ensuing acute hemolytic transfusion reaction [3]. Typing for *Mik* is no longer available due to depletion of the stock of serum containing anti‐*Mik* alloantibodies. More recently, 5 novel feline erythrocyte antigens (FEA), referred to as FEA 1–5, were identified based on the presence of naturally occurring alloantibodies [13]. Among 258 cats, 216 cats (84%) tested positive for FEA 1. Administration of FEA 1‐positive blood to 2 transfusion‐naïve FEA 1‐negative cats that did not have naturally occurring anti‐FEA 1 alloantibodies resulted in the development of anti‐FEA 1 alloantibodies as early as 5 days post‐transfusion, with one cat having detectable antibodies for over 400 days [14]. Development of an acute hemolytic transfusion reaction in a transfusion‐naïve type A cat subsequently determined to have naturally occurring anti‐FEA 1 alloantibodies after receiving type A and FEA 1‐positive blood highlights the clinical importance of naturally occurring anti‐FEA 1 alloantibodies in cats and lends support for the practice of performing a pre‐transfusion blood crossmatch in transfusion‐naïve cats [14]. Although rarely reported, another potential benefit of a blood crossmatch for transfusion‐naïve cats is identification of cats that have been mistyped by in‐clinic blood typing kits [15, 16].

There is no gold standard blood crossmatch method for cats, but several laboratory and in‐clinic methods have been used to evaluate RBC compatibility in this species: slide agglutination [2, 8], standard (i.e., not anti‐feline antiglobulin [AFG]‐enhanced) laboratory tube agglutination [3, 4, 6, 17], AFG‐enhanced laboratory tube agglutination [18], standard laboratory gel column [3, 6, 7, 13, 14], in‐clinic standard gel tube [8, 18], in‐clinic AFG‐enhanced gel column [7, 16], and a microtitration system [19]. The methods used most commonly by veterinary academic institutions and reference laboratories are the standard laboratory tube and gel column crossmatches. However, there is limited published information comparing results of these 2 crossmatch methods in cats [3, 6]. In the only feline study comparing a similar crossmatch methodology without and with AFG‐enhancement, a standard laboratory gel column and an in‐clinic AFG‐enhanced gel column, the latter revealed additional non‐A‐B incompatibilities that were not detected with the standard laboratory gel column crossmatch [7]. Finally, given the time and technical expertise required to perform laboratory tube and gel column crossmatches, as well as the potential for interobserver variation in interpretation, availability of a feline in‐clinic crossmatch kit that compares well to laboratory methods could be beneficial in an emergency setting.

The main objectives of this prospective study were to determine if: (1) there is agreement between results of feline crossmatches performed using the laboratory tube and gel column methods; (2) the addition of AFG increases detection of RBC incompatibilities using the laboratory tube and gel column methods compared to crossmatches performed without AFG enhancement; and (3) there is agreement between an in‐clinic AFG‐enhanced immunochromatographic strip crossmatch and the laboratory tube and gel column methods, both with and without AFG enhancement. A secondary objective was to evaluate interobserver variation in the interpretation of crossmatch results for the tube, gel column, and immunochromatographic strip methods.

## 2 Materials and Methods

### 2.1 Blood Samples

Blood samples (fresh [≤ 7 days of storage] EDTA‐anticoagulated blood and serum) used in this study as “recipient” and “donor” were obtained from previously transfused client‐owned cats after obtaining informed written consent and from surplus samples in the clinical laboratory, with a focus on, but not exclusively, previously transfused cats. In addition, EDTA‐anticoagulated blood (2 mL) was obtained from the hospital's feline blood donors to serve as a source of fresh RBCs for “donor” samples. If a cat's blood type was unknown, the type was determined using an immunochromatographic strip method (Quick Test BT A + B, Alvedia, Limonest, France). For client‐owned cats, the study protocol was approved by the university's Institutional Animal Care and Use Committee (protocol 807006) and the hospital's Privately Owned Animal Protocol Committee (protocol 605). For the hospital's feline blood donors, the study protocol was approved by the university's Institutional Animal Care and Use Committee (protocol 807012).

### 2.2 Blood Crossmatch Procedures

For a given recipient‐donor pair, major crossmatches were performed using a laboratory tube method without and with a polyclonal AFG (ImmunO; MP Biomedicals, Solon, OH), hereafter referred to as TUBE and AFG‐TUBE, respectively; a laboratory gel column (ID‐Micro Typing System Gel Test Cards, Ortho‐Clinical Diagnostics, Raritan, NJ) without and with AFG, hereafter referred to as GEL and AFG‐GEL, respectively; and an AFG‐enhanced immunochromatographic strip (Lab. Test XM, Alvedia), hereafter referred to as AFG‐STRIP. In addition, recipient auto‐controls were performed for TUBE and GEL crossmatches, both without and with AFG. Donor RBCs were washed in phosphate‐buffered saline (PBS) 3 times for both the TUBE and GEL crossmatch methods.

The TUBE crossmatch was performed following our hospital's clinical laboratory protocol. Briefly, recipient serum (50 μL) and donor 5% RBC suspension (25 μL) were placed into labeled tubes, mixed gently, and incubated at 37°C for 15 min, followed by centrifugation (750 *g* for 15 s). The RBC button was gently resuspended and examined for macroscopic agglutination, which was graded as previously described [3]. For the AFG‐TUBE crossmatch protocol, the RBCs from the TUBE crossmatch were washed 3 times with buffered saline to remove unbound antibodies, with the final wash completely decanted before adding AFG reagent (50 μL, diluted 1:2 with buffered saline). The tubes were incubated for 5 min at room temperature, centrifuged (750 *g* for 15 s), and examined for macroscopic agglutination, as before.

For the GEL crossmatch, a 0.8% RBC suspension was prepared by adding 10 μL washed pRBCs to 1 mL of low ionic strength saline solution (LISS; ID‐Micro Typing System MTS Diluent 2, Ortho‐Clinical Diagnostics). Serum (25 μL) and then RBC suspension (50 μL) were pipetted onto the top of appropriate gel columns: auto‐control, recipient serum + recipient RBCs; and major crossmatch, recipient serum + donor RBCs. Gel column cards were incubated (ID‐incubator 37 SI, DiaMed‐ID Micro Typing System, Cressier sur Morat, Switzerland) at 37°C for 15 min and then centrifuged (ID‐centrifuge 12 S II, DiaMed‐ID Micro Typing System) for 10 min. The agglutination was graded as previously described [7]. For the AFG‐GEL crossmatch, the appropriate 0.8% RBC suspension (50 μL) and serum (50 μL) were mixed in a tube and incubated at 37°C for 15 min. Following incubation, the RBC suspension was washed in PBS twice to remove free antibodies, with the final wash completely decanted before adding 15 μL PBS to resuspend the RBC pellet, which was then pipetted onto the top of the gel column, followed by 10 μL AFG (diluted 1:2 in buffered saline). The gel column card was incubated at 37°C for 5 min and centrifuged for 10 min, and agglutination was graded, as before.

The AFG‐STRIP crossmatch was performed as instructed by the kit's manufacturer. Donor pRBCs (10 μL, obtained from centrifuged EDTA‐anticoagulated blood tube via blood collector strip provided in kit) were mixed in a 5 mL test tube containing 160 μL of buffer A. Recipient serum (120 μL) was added to the donor RBC/buffer mixture, mixed gently, and incubated at room temperature for 10 min. The RBC suspension was washed 3 times with wash buffer (3 mL), with the supernatant discarded after each wash. Buffer B (80 μL) was added to the tube and gently mixed. Immunochromatographic test strips were placed into the tube for up to 5 min, allowing for sample migration up the strip to the control line, which was required to be visible for valid results. The crossmatch was deemed incompatible if a red line, even if weak, appeared at the XM site on the strip.

### 2.3 Interobserver Variation

All crossmatch results were independently scored by the primary study author (VKD, an internal medicine resident with no prior crossmatch experience), two experienced clinical laboratory technicians (SF and RG, with 16 and 5 years, respectively, of experience performing and interpreting crossmatches), and one board‐certified clinical pathologist (NMW, with 21 years of experience in blood compatibility testing). All observers, except the primary author, were blinded as to the identification of the samples. The primary author scored and recorded all crossmatch results immediately after completion. Results of all TUBE crossmatches were recorded by video to capture the resuspension of the RBC button after centrifugation. All GEL and AFG‐STRIP crossmatches were photographed immediately after centrifugation and the final 5 min incubation step, respectively. If all 4 observers deemed a crossmatch incompatible, the score reported is the median score of the 4 observers. If 3 of 4 observers deemed a crossmatch incompatible, the score reported is the median score of the 3 observers. If only 2 of 4 observers deemed a crossmatch incompatible, the clinical pathologist's interpretation was given priority.

### 2.4 Statistical Analysis

A two‐sample comparison of proportion test was used to determine the number of compatible crossmatches needed to detect a proportion of 88% compatible samples using the gel method, compared to presumed 100% detection of compatible samples using the tube method, as previously reported [20]. This calculation resulted in a required sample size of 77 compatible crossmatches for each method. To detect a similar difference in proportions of incompatible samples, 77 incompatible crossmatches are also needed for each method. Additional assumptions included a power of 0.8 and type I error rate of 0.05.

Most continuous variables were not normally distributed as determined visually and by the skewness and kurtosis tests for normality [21]. Therefore, results are reported as median (and range). The chi‐square or Fisher's exact test was employed to examine the relationship between two categorical variables, depending on whether the number of observations per cell was greater than 5 or not. The Spearman correlation was used to assess the correlation between continuous variables, such as incompatibility scores. Simple logistic regression was used to investigate the correlation between binary outcomes (such as compatible or incompatible crossmatch test results) and continuous predictor variables (such as degree of incompatibility). The kappa statistic was calculated to determine agreement between compatibility and incompatibility scoring by different observers, with Kappa values (*κ*) of 0 and 1 indicating random and perfect agreement, respectively. For intermediate values, the following interpretations were used: < 0, poor agreement; 0–0.2, slight agreement; 0.21–0.4, fair agreement; 0.41–0.60, moderate agreement; 0.61–0.80, substantial agreement; and 0.81–1.0, almost perfect agreement. A *p* value of < 0.05 was considered significant for all tests. All statistical evaluations were performed using a statistical software package (Stata 14.0 for Mac, Stata Corporation, College Station, TX).

## 3 Results

### 3.1 Blood Samples

Major crossmatches were performed using serum from 68 “recipient” cats and RBCs from 44 “donor” cats. Each “recipient” serum sample was crossmatched to RBCs from a median of 3 “donors” (range, 1–10), resulting in a total of 211 “recipient‐donor” pairs. Among the “recipient” cats, 61 had blood type A, 6 had blood type B, and 1 had blood type AB. “Donor” cats included 42 with blood type A and 2 with blood type B. Fifteen “recipient” cats were transfusion‐naïve, and 53 “recipient” cats had been transfused days to several years earlier.

### 3.2 Blood Crossmatch Procedures

#### 3.2.1 Tube Crossmatch

“Recipient” auto‐controls were negative, that is, there was no evidence of RBC agglutination in the tubes without or with the addition of AFG. A total of 211 TUBE and 210 AFG‐TUBE crossmatches were performed. RBC incompatibilities (median 2+; range, 1+ to 4+) were noted in 35 (17%) of the TUBE crossmatches, which included serum from 26 “recipient” cats, 4 of which were transfusion naïve, and RBCs from 28 “donor” cats. Of the 35 TUBE crossmatches that demonstrated incompatibility, 32 included a “recipient” and “donor” that were both blood type A. There were 3 type‐mismatched pairs, including 2 type B “recipients” that were incompatible with 1 or 2 type A “donors” (Table 1). RBC incompatibilities (median 2+; range, 1+ to 3.5+) were noted in 29 (14%) AFG‐TUBE crossmatches, which included serum from 21 “recipient” cats, 3 of which were transfusion naïve, and RBCs from 23 “donor” cats. Of the 29 AFG‐TUBE crossmatches that demonstrated incompatibility, 25 included a “recipient” and “donor” that were both blood type A. There were 4 type‐mismatched pairs, including 2 type B “recipients” that were each incompatible with 2 different type A “donors” (Table 1). For the determination of the degree of incompatibility, there was a moderate correlation between TUBE and AFG‐TUBE crossmatches (*ρ* = 0.51; *p* < 001; Table 2). Among 46 “recipient‐donor” pairs that demonstrated incompatibility on either the TUBE or AFG‐TUBE crossmatch or both, 12 pairs demonstrated incompatibility only with AFG‐enhancement (Table 2).

**TABLE 1:** Major crossmatch results for blood type B and AB recipients and both A‐B matched and mismatched donors.

Recipient			Donor	Crossmatch results				
ID	Prior transfusion	Blood type	Blood type	TUBE	AFG‐TUBE	GEL	AFG‐GEL	AFG‐STRIP
1	No	B	B	0	1+	0	0	0
2	Yes	B	B	1+	0	2.5+	0	0
			A	0	3+	3+	Lysed	1
			A	4+	3+	3+	3.5+	1
3	No	B	B	0	0	0	0	0
4	Yes	B	B	0	0	3+	0	0
5	Yes	B	B	0	0	0	0	0
6	Yes	B	A	4+	3+	4+	4+	1
			A	4+	3+	4+	4+	1
7	No	AB	A	0	0	3+	0	0
			A	0	0	4+	0	0

Abbreviations: AFG, anti‐feline antiglobulin enhanced; AFG‐GEL, AFG‐enhanced laboratory gel column crossmatch; AFG‐STRIP, AFG‐enhanced immunochromatographic strip crossmatch; AFG‐TUBE, AFG‐enhanced laboratory tube crossmatch; GEL, standard laboratory gel column crossmatch; score 0, compatible; scores 1–4, incompatible (for AFG‐STRIP, grading of incompatibility is not possible, i.e., result is either compatible [0] or incompatible [1]); TUBE, standard laboratory tube crossmatch.

**TABLE 2:** Number and percentages of compatible and incompatible major crossmatch results for 210 recipient‐donor pairs in standard compared to AFG‐enhanced TUBE and GEL crossmatches.

Crossmatch method	Both standard and AFG‐enhanced crossmatches compatible	Only standard crossmatch incompatible	Only AFG‐enhanced crossmatch incompatible	Both standard and AFG‐enhanced crossmatches incompatible	Total number of crossmatches	*p* ^a^
TUBE	164 (78%)	17 (8%)	12 (6%)	17 (8%)	210	< 0.001
GEL	97 (46%)	46 (22%)	22 (11%)	45 (21%)	210	< 0.001

Abbreviations: AFG, anti‐feline globulin; GEL, laboratory gel column crossmatch; TUBE, laboratory tube crossmatch.

^a^ Correlation regarding degree of incompatibility between standard and AFG‐enhanced crossmatches for each method as determined by Spearman correlation.

#### 3.2.2 Laboratory Gel Column Crossmatch

Recipient auto‐controls were negative, that is, there was no evidence of RBC agglutination in the gel column without or with the addition of AFG. A total of 211 GEL and 210 AFG‐GEL crossmatches were performed. RBC incompatibilities (median, 3.25+; range, 1+ to 4+) were noted in 92 (44%) of the GEL crossmatches using serum from 42 “recipient” cats, 5 of which were transfusion naïve, and RBCs from 37 “donor” cats. Of the 92 GEL crossmatches that demonstrated incompatibility, 86 included a “recipient” and “donor” that were both blood type A. There were 6 type‐mismatched pairs, including 2 type B “recipients” and 1 type AB “recipient” that were each incompatible with 2 different type A “donors” (Table 1). There were 67 (32%) AFG‐GEL incompatible crossmatches, which included serum from 30 “recipient” cats, 2 of which were transfusion naïve, and RBCs from 33 “donor” cats. Of the 67 AFG‐GEL crossmatches that demonstrated incompatibility, 63 included a “recipient” and “donor” that were both blood type A. There were 4 type‐mismatched pairs, including 2 type B “recipients” that were each incompatible with 2 different type A “donors” (Table 1). As for the tube crossmatch, a corresponding AFG‐GEL crossmatch was inadvertently not performed for one of the GEL crossmatches. For determination of the degree of incompatibility, there was a fair correlation between GEL and AFG‐GEL crossmatches (*ρ* = 0.4; *p* < 001; Table 2). One of the type‐mismatched AFG‐GEL incompatible crossmatches performed using serum from a type B “recipient” and type A “donor” could not be assigned a compatibility score since all “donor” RBCs were lysed. The corresponding GEL crossmatch yielded a 3+ incompatibility. Among 113 recipient‐donor pairs that demonstrated incompatibility on either the GEL or AFG‐GEL crossmatch or both, 22 pairs demonstrated incompatibility only with AFG‐enhancement (Table 2).

#### 3.2.3 Immunochromatographic Strip Method

The AFG‐STRIP crossmatch was performed for 133 recipient‐donor pairs. RBC incompatibility was noted on 15 strips (11%), which used serum from 9 “recipient” cats, all previously transfused, and RBCs from 12 “donor” cats. Of the 15 AFG‐STRIP crossmatches that demonstrated incompatibility, 11 included a “recipient” and “donor” that were both blood type A. There were 4 type‐mismatched pairs, including 2 type B “recipients” that were each incompatible with 2 different type A “donors” (Table 1).

### 3.3 Comparison of Crossmatch Methods

In a comparison of 211 TUBE and GEL crossmatches, there was a fair correlation (*ρ* = 0.21; *p* = 0.002; Table 3) for determining the degree of incompatibility. With the addition of AFG to 210 TUBE and GEL crossmatches, there was a moderate correlation (*ρ* = 0.41; *p* < 0.001; Table 3) for determining the degree of incompatibility. A total of 123 of 211 (58%) recipient‐donor pairs were deemed incompatible based on 1 or more crossmatch methods (Table S1). The AFG‐STRIP crossmatch was performed for 87 of the 123 recipient‐donor pairs deemed incompatible by the TUBE and/or GEL crossmatch. RBC incompatibilities were identified in 15 recipient‐donor pairs (17%), and 14 of these 15 RBC incompatibilities were also observed in both the TUBE and GEL crossmatches, without and/or with AFG‐enhancement (Table 4). AFG‐STRIP crossmatch tests were read as compatible or incompatible, as the line on the test strip does not lend itself to grading the strength of incompatibility. Therefore, comparisons of the AFG‐STRIP to other crossmatch methods were performed for the determination of compatibility rather than the degree of incompatibility. When comparing TUBE and AFG‐TUBE crossmatches to the AFG‐STRIP crossmatch, there was a correlation between these methods for determining compatibility (*p* = 0.001 and *p* < 0.001, respectively; Table 4). Each unit increase in the degree of incompatibility (e.g., 1+ vs. 2+) detected in a tube or gel crossmatch increased the odds of detecting a RBC incompatibility using the AFG‐strip method. For the TUBE and AFG‐TUBE crossmatches, the odds were 2.9 times greater (95% CI 1.8–4.5, *p* < 0.001) and 6.1 times greater (95% CI 3.1–11.8, *p* < 0.001), respectively, that the AFG‐STRIP would identify a RBC incompatibility. For GEL and AFG‐GEL crossmatches, the odds were 1.5 times greater (95% CI 1.1–2.2, *p* < 0.02) and 4.6 times greater (95% CI 2.1–10.6, *p* < 0.001), respectively, that the AFG‐STRIP would identify a RBC compatibility.

**TABLE 3:** Number of compatible and incompatible major crossmatch results for TUBE and AFG‐TUBE compared to GEL and AFG‐GEL, respectively.

	GEL compatible	GEL incompatible	Total number of crossmatches	*p* ^a^
TUBE compatible	107	69	176	
TUBE incompatible	12	23	35	0.002
Total number of crossmatches	119	92	211	

	AFG‐GEL compatible	AFG‐GEL incompatible		
AFG‐TUBE compatible	135	46	181	
AFG‐TUBE incompatible	8	21	29	< 0.001
Total number of crossmatches	143	67	210	

Abbreviations: AFG, anti‐feline globulin; AFG‐GEL, AFG‐enhanced laboratory gel column crossmatch; AFG‐TUBE, AFG‐enhanced laboratory tube crossmatch; GEL, standard laboratory gel column crossmatch; TUBE, standard laboratory tube crossmatch.

^a^ Correlation between two crossmatch methods for determination of degree of incompatibility as determined by Spearman correlation.

**TABLE 4:** Number of compatible and incompatible major crossmatch results using the AFG‐STRIP compared to standard and AFG‐enhanced TUBE and GEL crossmatches.

	AFG‐STRIP compatible	AFG‐STRIP incompatible	Total number of crossmatches	*p* ^a^
TUBE compatible	92	5	97	
TUBE incompatible	26	10	36	0.001
Total number of crossmatches	118	15	133	
AFG‐TUBE compatible	102	1	103	
AFG‐TUBE incompatible	15	14	29	< 0.001
Total number of crossmatches	117	15	132	
GEL compatible	63	3	66	
GEL incompatible	55	12	67	0.026
Total number of crossmatches	118	15	133	
AFG‐GEL compatible	79	0	79	
AFG‐GEL incompatible	38	15	53	< 0.001
Total number of crossmatches	117	15	132	

Abbreviations: AFG, anti‐feline globulin; AFG‐GEL, AFG‐enhanced laboratory gel column crossmatch; AFG‐STRIP, AFG‐enhanced immunochromatographic strip crossmatch; AFG‐TUBE, AFG‐enhanced laboratory tube crossmatch; GEL, standard laboratory gel column crossmatch; TUBE, standard laboratory tube crossmatch.

^a^ Correlation between two crossmatch methods for determination of compatibility as determined by Fisher's exact test.

### 3.4 Interobserver Variation

A total of 421 tube crossmatches (including both standard and AFG‐enhanced), 420 gel columns (including both standard and AFG‐enhanced, minus the single AFG‐GEL crossmatch that resulted in complete hemolysis), and 133 AFG‐STRIP crossmatches were interpreted by 4 individuals. For the tube crossmatch, there was substantial agreement (*κ* = 0.7, *p* < 0.001) among the 4 observers for determining compatibility (i.e., agglutination score of 0) and moderate agreement (*κ* = 0.5, *p* < 0.001) for scoring incompatibility (i.e., scores of 1 to 4+). The measure of agreement for the tube crossmatch was significantly different from random agreement for all agglutination scores (*p* < 0.001). For the gel column crossmatch, there was almost perfect agreement (*κ* = 0.82, *p* < 0.001) among the 4 observers for determining compatibility and moderate agreement (*κ* = 0.57, *p <* 0.001) for scoring incompatibility. The measure of agreement for the gel column crossmatch was significantly different from random agreement for all agglutination scores (*p* < 0.001). For the AFG‐STRIP, there was almost perfect agreement (*κ* = 0.85, *p* < 0.001) for determining compatibility.

## 4 Discussion

Feline crossmatch testing lacks a gold standard, so it is not possible to conclude that one technique is superior for identifying clinically important RBC incompatibilities in cats. The data demonstrate that there is fair to moderate correlation between crossmatch methods for the determination of RBC incompatibilities, that is, between the TUBE and AFG‐TUBE, the GEL and AFG‐GEL, the TUBE and GEL, and the AFG‐TUBE and AFG‐GEL. For TUBE and GEL crossmatches, without and/or with AFG‐enhancement, concurrently compatible results were seen in 88 of 211 (42%) tests; concurrently incompatible results were seen in only 39 of 123 (32%). For corresponding recipient‐donor pairs, AFG‐enhancement of TUBE and GEL crossmatches resulted in the detection of RBC incompatibilities that were not initially observed in crossmatches lacking AFG enhancement. For both standard and AFG‐enhanced TUBE and GEL crossmatches, there was a correlation with the AFG‐STRIP crossmatch for determining compatibility. The odds of detecting an incompatibility with the AFG‐STRIP were greater with each unit increase in the incompatibility score for the TUBE and GEL crossmatches. Lastly, there was substantial to almost perfect agreement among 4 observers in determining compatibility with all crossmatch methods, as well as moderate agreement for scoring incompatibility with the TUBE and GEL crossmatch methods.

In two previous studies that included a limited comparison of standard TUBE and GEL crossmatches in cats, there was strong agreement for the determination of RBC compatibility between methods [3, 6]. Both TUBE and GEL crossmatches identified the same incompatible and compatible reactions in a survey study of *Mik* and other potential blood incompatibilities of blood donors, as well as between blood donors for a recipient with naturally occurring anti‐*Mik* alloantibodies [3]. In another study evaluating the prevalence of naturally occurring non‐AB RBC incompatibilities, results of both TUBE and GEL crossmatches were in agreement (*n* = 40 compatible, *n* = 6 incompatible) [6]. A discrepancy was noted for 1 recipient due to robust rouleaux formation that yielded a 4+ mixed field agglutination in the auto‐control and major crossmatch using the GEL method but not in the tube method [6]. In a study comparing TUBE and GEL methods to assess DEA 7 blood incompatibility in 40 dogs, TUBE and GEL results were concordant only when microscopic evaluation of the TUBE crossmatch was considered, where any degree of microscopic agglutination was considered positive [22]. Anti‐DEA 7 antibodies were reportedly weak, with 18 of 21 incompatible GEL crossmatches scored as 1+ agglutination and all corresponding TUBE crossmatches interpreted as positive based on hemolysis or microscopic agglutination, that is, none demonstrated macroscopic agglutination [22].

Our study included a comparison of TUBE and GEL crossmatch results for the standard (*n* = 211 recipient‐donor pairs) and AFG‐enhanced (*n* = 210 recipient‐donor pairs) tests. Despite the correlation between TUBE and GEL crossmatches for determination of the degree of incompatibility, one cannot conclude that the methods are equal in identifying RBC alloantibodies, as some recipient‐donor pairs were deemed incompatible by one method only. There are several possible explanations for the differences in results between crossmatch methods. Differences in RBC suspension diluents may alter antigen–antibody binding, that is, LISS in the GEL method and PBS in the TUBE method, as LISS may enhance binding by decreasing steric hindrance. Overzealous resuspension of the RBC button in the TUBE method could lead to misidentification of a result as compatible. Increased sensitivity of the GEL crossmatch may lead to the detection of non‐specific agglutination or weak RBC incompatibilities that may be clinically insignificant. Rouleaux formation may be misinterpreted as RBC agglutination in the GEL crossmatch. TUBE crossmatches were not evaluated microscopically and, therefore, saline dispersion was not performed to rule out rouleaux formation.

Addition of species‐specific antiglobulin to TUBE and GEL crossmatches, that is, AFG‐enhanced, has been reported to increase the detection of alloantibodies in dogs and cats [7, 23, 24]. Of 145 incompatible TUBE crossmatches in critically ill dogs, 67 (46.2%) were identified only after the addition of anti‐canine globulin (ACG) [23]. Similarly, in a study evaluating post‐transfusion RBC alloimmunization in dogs using a GEL crossmatch without and with ACG enhancement, 10 of 33 dogs had major crossmatch incompatibilities, with RBC agglutination noted in 4 of 10 dogs only with the addition of ACG [24]. In a study evaluating naturally occurring RBC alloantibodies in cats using a GEL and an in‐clinic AFG‐GEL crossmatch, both methods identified RBC incompatibilities in 102 recipient‐donor pairs, with an additional 15 incompatible pairings detected only with AFG enhancement [7]. In our study, RBC agglutination was noted only with the addition of AFG for 12 of 46 recipient‐donor pairs for the TUBE crossmatch and 22 of 113 recipient‐donor pairs for the GEL crossmatch. Similar to findings in a previous study comparing GEL and ACG‐GEL crossmatches in dogs [24], we observed RBC agglutination in some feline recipient‐donor pairs only in the absence of AFG enhancement, that is, only in the standard TUBE or GEL crossmatch. Potential explanations include dilution or removal of weakly bound RBC antibodies in the post‐incubation washing step, neutralization of AFG reagent by residual unbound antibodies as a result of inadequate washing, a prozone effect caused by excessive AFG interfering with RBC agglutination, and insufficient time for AFG to bind to RBC‐bound IgG before centrifugation of tubes and gel columns. Interestingly, false‐negative compatible anti‐human globulin crossmatches have been documented in samples from humans with well‐characterized alloantibodies intentionally crossmatched against donor RBCs expressing the cognate antigen, a finding attributed to the antibody titer (i.e., low titer is more likely to yield a false‐negative result) and donor cell zygosity (i.e., RBCs heterozygous rather than homozygous for antigen are more likely to yield a false‐negative result) [25].

Given the time, equipment, and technical expertise required to perform TUBE and GEL crossmatches, the availability of an in‐clinic crossmatch kit that can detect RBC incompatibilities in cats quickly and simply would be invaluable in providing safe and effective RBC transfusions. A study comparing an in‐clinic gel tube crossmatch kit to an AFG‐TUBE crossmatch for detection of RBC incompatibilities in cats revealed poor agreement (*κ* = 0.111) between methods, with 15 of 131 major crossmatches deemed incompatible by the in‐clinic gel tube kit and compatible by the AFG‐TUBE method and another 15 major crossmatches deemed incompatible by the AFG‐TUBE method and compatible by the in‐clinic gel tube kit [18]. In contrast, in a study comparing an in‐clinic AFG‐enhanced gel column kit and a GEL crossmatch for detection of naturally occurring RBC alloantibodies in cats, there was a strong correlation (*r* = 0.938) between methods, with complete agreement in determination of compatibility (*n* = 329) or incompatibility (*n* = 102) for all but 15 donor‐recipient pairings that were deemed incompatible only with AFG enhancement [7]. The authors are not aware of published studies evaluating the in‐clinic AFG‐STRIP crossmatch in cats, but in a study comparing a similar in‐clinic ACG‐STRIP and an ACG‐GEL crossmatch in dogs, results were concordant (*r* ^2^ = 0.96) between methods [26]. However, in a study comparing the same ACG‐STRIP to a TUBE crossmatch (without and with ACG), there was no agreement between methods; the ACG‐STRIP detected only 3 of 26 incompatibilities detected in the TUBE crossmatch [23]. Similarly, in another study, the ACG‐STRIP detected only 1 of 21 DEA 7‐incompatible crossmatches identified with the GEL and TUBE crossmatches [22].

Potential benefits of the AFG‐STRIP crossmatch include its simple methodology, time efficiency (25 min, as per the manufacturer), lack of need for specialized equipment, and ease of interpreting results. Instructions provided with the AFG‐STRIP crossmatch indicate that it is mandatory to perform blood typing before crossmatching. However, in our study, we opted to include 4 A‐B‐mismatched recipient (B)‐donor (A) pairs that would be expected to yield RBC incompatibilities across all crossmatch methods. Indeed, the TUBE and GEL crossmatches, as well as the AFG‐STRIP crossmatches, were all deemed incompatible for all A‐B‐mismatched pairs. Given that 14 of 15 RBC incompatibilities identified with the AFG‐STRIP were also observed in both the TUBE and GEL crossmatches, without and/or with AFG‐enhancement, incompatibilities noted with the in‐clinic kit were likely true positives. There were no recipient‐donor pairings that were incompatible only with the AFG‐STRIP, suggesting that false‐positive RBC incompatibilities are unlikely with the use of this in‐clinic kit. However, there were 25 recipient‐donor pairings with RBC incompatibilities noted in both the TUBE and GEL crossmatches, without or with AFG‐enhancement, that were deemed compatible with the AFG‐STRIP. Therefore, the in‐clinic kit is less likely to detect weaker RBC incompatibilities, supported by our data analysis indicating that the odds of detecting an RBC incompatibility using the AFG‐STRIP method were greater for each unit increase in the degree of incompatibility detected by the TUBE and GEL crossmatches, without or with AFG‐enhancement.

Interpretation of crossmatch results is somewhat subjective with respect to determining compatibility and grading the degree of incompatibility. Of the 3 crossmatch methods evaluated in our study, the AFG‐STRIP had the least variability in interpretation of compatibility among 4 observers, with almost perfect agreement (85%). Given that RBC incompatibility is determined by the observer detecting a red line at the test site on the immunochromatographic strip, it is not surprising that the interpretation of this in‐clinic crossmatch method is more straightforward than the laboratory methods. It has been suggested that the interpretation of the GEL crossmatch is more standardized than that of the TUBE crossmatch [3, 7]. Our results support this assumption, with substantial agreement (70%) and almost perfect agreement (82%) among 4 observers in determining compatibility in 421 TUBE and 420 GEL crossmatches, respectively. For both the TUBE and GEL crossmatch methods, there was moderate agreement (50% and 57%, respectively) for scoring the degree of incompatibility. While we are not aware of published studies evaluating interobserver variation for interpretation of TUBE crossmatches for determination of RBC compatibility in veterinary species, previous studies evaluating interobserver (*n* = 3–4) variation for the GEL crossmatch method have documented near perfect agreement (94%–100%) for determining compatibility and moderate to strong agreement (76%–97%) for scoring of incompatibility in dogs and horses [24, 27, 28]. Results of our published studies highlight the subjectivity of the grading systems for both the TUBE and GEL crossmatches, even among experienced observers. However, variability in the interpretation of the degree of incompatibility is less important than agreement in determining compatibility in a clinical situation, given that the goal is always to administer compatible RBCs, if available.

Limitations of our study include the lack of a gold standard feline crossmatch method to which our results could be compared. Also, microscopic evaluation for rouleaux formation in TUBE crossmatches with weak (1+) RBC agglutination was not performed due to a limited serum sample volume for “recipients” that precluded setting up 2 TUBE crossmatches for each recipient‐donor pair to allow for both microscopic evaluation and the antiglobulin phase of the crossmatch. AFG‐STRIP crossmatches were not performed for all 211 donor‐recipient pairs due to a limited supply of strips, so priority was given to evaluating AFG‐STRIP crossmatch for donor‐recipient pairs identified to be incompatible in the TUBE or GEL crossmatches, resulting in potential bias by the primary study author but not the other 3 observers who were blinded to sample identification and results of any corresponding TUBE or GEL crossmatches. As with most crossmatch studies, we could not determine the clinical relevance of RBC incompatibilities noted with any of the crossmatch methods evaluated. Also, TUBE crossmatches were evaluated by 3 of 4 observers via video recording of resuspension of RBC pellet since it was impractical for all 4 reviewers to do so in real time.

In conclusion, although the aim of our study was not to determine the gold standard method for feline crossmatches, we demonstrated that some RBC incompatibilities are evident in only 1 crossmatch method, that is, the TUBE or GEL crossmatch, and only with the addition or in the absence of AFG. Without being able to determine the clinical relevance of any of the observed RBC incompatibilities (other than the known A‐B mismatches), it is not possible to conclude that one method is superior to another for feline crossmatches. While the in‐clinic AFG‐STRIP crossmatch is simple and quick to perform and interpret, it might not be sufficiently sensitive to detect weaker RBC incompatibilities.

## Conflicts of Interest

The authors declare no conflicts of interest.

## Supporting information


**Table S1:** vcp70039‐sup‐0001‐TableS1.docx.
