# Supplementary material for: Comparison of Three Feline Crossmatch Methods—Tube, Gel Column, and Immunochromatographic Strip
Source: Vet Clin Pathol. 2025 Aug 22;54(3):230–8. doi: 10.1111/vcp.70039 (PMC12444005; doi:10.1111/vcp.70039)
Supplement: Supplementary file 1 — Table S1: vcp70039‐sup‐0001‐TableS1.docx. [file VCP-54-230-s001.docx]

**Supplementary table 1**. Number of RBC incompatibilities detected with one or more crossmatch methods

| **Crossmatch methods with RBC incompatibilities detected** | **Number of recipient-donor pairs with RBC incompatibilities detected** | **Number of crossmatches that did not include AFG-STRIP** |
| --- | --- | --- |
| TUBE only | 6 | 3 |
| AFG-TUBE only | 5 | 2 |
| TUBE and AFG-TUBE only | 0 | - |
| GEL only | 37 | 15 |
| AFG-GEL only | 17 | 7 |
| GEL and AFG-GEL only | 18 | 5 |
| TUBE and GEL only | 3* | 1 |
| AFG-TUBE and AFG-GEL only | 2 | - |
| TUBE, AFG-TUBE, and GEL only | 2 | - |
| AFG-TUBE and GEL only | 3 | 1 |
| TUBE and AFG-GEL only | 2 | 1 |
| TUBE, GEL, and AFG-GEL only | 6 | - |
| TUBE, AFG-TUBE, GEL, and AFG-GEL only | 7 | 1 |
| TUBE, AFG-TUBE, AFG-GEL, and AFG-STRIP only | 3 | - |
| AFG-TUBE, GEL, AFG-GEL, and AFG-STRIP only | 4# | - |
| TUBE, AFG-TUBE, GEL, AFG-GEL, and AFG-STRIP | 7¶ | - |
| GEL, AFG-GEL, and AFG-STRIP only | 1 | - |
| **Total** | 123 | 36 |

* Anti-feline antiglobulin enhancement inadvertently omitted for 1 donor-recipient pair

# One recipient-donor pair was AB mismatched (type B recipient, type A donor)

¶ Three recipient-donor pairs were AB mismatched (type B recipient, type A donor)
